# Cumulative Vascular Risk Factor Burden and Anxiety–Depression Symptoms Co-Occurrence: A Cross-Sectional Study

**DOI:** 10.3390/brainsci16101058

**Published:** 2026-09-30

**Authors:** Sisi Peng, Bingqian Lv, Wenyan Zhou, Linna Ji, Junjian Zhang

**Affiliations:** 1 Department of Neuropsychology, Zhongnan Hospital of Wuhan University, Wuhan 430071, China; pengsisi@whu.edu.cn; 2 Hubei Provincial Clinical Research Center for Dementia and Cognitive Impairment, Wuhan 430071, China; linnaji@whu.edu.cn; 3 Department of Neurology, Zhongnan Hospital of Wuhan University, No. 169 East Lake Road, Wuchang District, Wuhan 430071, China; 2020305232011@whu.edu.cn (B.L.); zhouwenyan@whu.edu.cn (W.Z.)

**Keywords:** vascular risk factor burden, anxiety–depression symptom co-occurrence, anxiety symptoms, depressive symptoms, emotional symptoms, mental health

## Abstract

**Highlights:**

**What are the main findings?**

- Higher vascular risk factor burden was associated with greater anxiety and depressive symptom severity.
- Higher vascular risk factor burden was also associated with greater odds of anxiety–depression symptom co-occurrence after multivariable adjustment.

**What are the implications of the main findings?**

- Vascular risk factor burden may provide useful clinical context for recognizing individuals with greater emotional symptom burden.

**Abstract:**

**Background:** Individuals with vascular risk factors (VRFs) often experience underrecognized emotional symptoms. This study examined the association of vascular risk factor burden (VRF burden) with anxiety, depression and their symptom co-occurrence. **Methods:** A total of 381 participants were classified by HAMA and HAMD-17 scores into control, anxiety, depression, and anxiety–depression symptom co-occurrence groups. VRF burden was defined as the number of eight prespecified VRFs. Multivariable linear and logistic regression analyses were performed. **Results:** Anxiety–depression symptom co-occurrence was the most common symptom pattern (40.9%). Greater VRF burden was associated with higher HAMA (B = 2.549, 95% CI: 2.028–3.070) and HAMD-17 scores (B = 1.802, 95% CI: 1.258–2.346; both *p* < 0.001). Greater VRF burden was also associated with higher odds of symptom co-occurrence after multivariable adjustment (adjusted OR = 1.944, 95% CI: 1.548–2.441, *p* < 0.001). **Conclusions:** VRF burden was associated with more severe emotional symptoms and higher odds of anxiety–depression symptom co-occurrence. Prospective studies are needed to clarify temporal relationships.

## 1. Introduction

Anxiety and depressive symptoms represent a substantial global health burden and are associated with impaired functioning, reduced quality of life, and considerable societal impact [1,2]. Although emotional symptoms have traditionally been considered within the domain of mental health, accumulating evidence indicates that they are also associated with vascular risk factors (VRFs), including hypertension, diabetes, hyperlipidemia, and smoking, suggesting an important interplay between mental and vascular health. Epidemiological studies have shown that VRFs and cardiovascular disease are associated with increased anxiety and depressive symptom burden [3,4,5]. Recent studies further suggest that vascular risk factor burden (VRF burden), reflecting the cumulative burden of multiple VRFs, may provide a more comprehensive characterization of the overall vascular risk profile than individual risk factors [6,7].

The relationship between vascular factors and emotional symptoms may reflect shared biological pathways involving inflammation, endothelial dysfunction, oxidative stress, and altered cerebrovascular function [8,9,10]. However, emotional symptoms are multifactorial, and these mechanisms alone may not fully explain individual differences in emotional vulnerability. Based on clinical and neurobiological evidence linking vascular conditions with depressive symptoms, Alexopoulos et al. proposed the “vascular depression” hypothesis, suggesting that cerebrovascular burden may be associated with alterations in mood-related neural circuits and may be associated with greater vulnerability to depressive symptoms [11]. This framework, however, was developed primarily for depression, and its relevance to anxiety symptoms or anxiety–depression symptom co-occurrence is less well established. Thus, whether cumulative vascular burden is similarly associated with anxiety-related symptom patterns remains uncertain.

In clinical practice, VRFs rarely occur in isolation but often cluster within individuals. Given the cumulative effects of multiple VRFs, assessing individual risk factors alone may not fully capture an individual’s overall vascular risk profile. VRF burden has therefore been increasingly used to characterize cumulative vascular risk. Large cohort and neuroimaging studies have shown that higher VRF burden is associated with white matter changes, brain atrophy, and other markers of vascular-related brain injury [12,13,14]. However, most previous studies have focused on cognitive and neuroimaging outcomes, while the relationship between cumulative VRF burden and emotional symptoms, particularly anxiety–depression symptom co-occurrence, remains insufficiently characterized [15,16,17,18].

Anxiety and depressive symptoms frequently co-occur, and their combination may be associated with greater symptom severity, poorer psychosocial functioning, and higher clinical burden than either symptom domain alone [19,20]. Given that the vascular basis of anxiety is less established than that of depression, examining symptom co-occurrence may help clarify whether cumulative vascular burden is associated with a broader emotional symptom profile.

To address this gap, the present study investigated the associations of VRF burden with anxiety symptoms, depressive symptoms, and anxiety–depression symptom co-occurrence among middle-aged and older adults with VRFs. We hypothesized that higher VRF burden would be associated with greater severity of anxiety and depressive symptoms, as well as a higher likelihood of anxiety–depression symptom co-occurrence. By focusing on VRF burden rather than individual risk factors, this study aimed to further characterize the relationship between vascular health and emotional symptoms and to explore the potential relevance of considering emotional symptoms in the clinical assessment of individuals with VRFs.

## 2. Materials and Methods

### 2.1. Study Population

This cross-sectional study consecutively enrolled individuals with at least one VRF who attended the Memory Clinic of the Department of Neuropsychology, Zhongnan Hospital of Wuhan University, between January 2024 and January 2025.

The inclusion criteria were as follows: (1) age ≥ 50 years; (2) presence of at least one VRF, including hypertension, diabetes, hyperlipidemia, CHD, physical inactivity (PI), current smoking, history of cerebral infarction (CI), or transient ischemic attack (TIA); (3) ability to complete the required neuropsychological assessments; and (4) provision of written informed consent. The exclusion criteria were as follows: (1) a previous diagnosis of severe psychiatric disorders, such as schizophrenia or bipolar disorder; (2) severe cognitive impairment, aphasia, or disturbance of consciousness that precluded completion of the assessments; (3) acute infection, malignant tumor, or severe hepatic or renal dysfunction; and (4) incomplete clinical data.

A total of 498 individuals were initially assessed. Of these, 7 did not meet the inclusion criteria and 110 were excluded because of incomplete data, including missing HAMA and/or HAMD-17 assessments (n = 78), missing both scale and medical history information (n = 14), or missing medical history information only (n = 18). Finally, 381 participants were included in the analysis.

### 2.2. Clinical Data Collection

Demographic and clinical data were collected, including age, sex, marital status, years of education, body mass index (BMI), lifestyle factors, and medical history. Lifestyle factors included current smoking status and physical activity level. Medical history included hypertension, diabetes, hyperlipidemia, CHD, CI, and TIA. Height and weight were measured, and BMI was calculated as weight (kg) divided by height squared (m^2^).

Individual VRFs included hypertension, diabetes, hyperlipidemia, CHD, TIA, CI, current smoking, and PI. VRF burden was calculated as the total number of present VRFs, with each factor assigned one point. The theoretical score range among eligible participants was 1–8, while the observed range in the present study was 1–7. Higher scores indicated greater VRF burden. Hypertension, diabetes, hyperlipidemia, and CHD were ascertained primarily from documented diagnoses in the hospital medical records and/or current use of corresponding medications. When available, relevant clinical measurements and laboratory data, including blood pressure, fasting glucose or HbA1c, and lipid profiles, were also reviewed to support the recorded diagnoses. Thus, classification of these VRFs was based on a combination of medical history, medication records, and available clinical or laboratory information rather than on self-report alone. Current smoking was defined as regular smoking within the previous 12 months. PI was defined according to the World Health Organization recommendations as not meeting the recommended level of physical activity (≥150 min of moderate-intensity activity per week, ≥75 min of vigorous-intensity activity per week, or an equivalent combination) [21].

### 2.3. Neuropsychological Assessment and Group Classification

Anxiety symptoms were assessed using the Chinese version of Hamilton Anxiety Rating Scale (HAMA), and depressive symptoms were assessed using the Chinese version of the 17-item Hamilton Depression Rating Scale (HAMD-17) [22,23]. All assessments were administered by one trained neuropsychological assessor who had received standardized instruction prior to the study. Data were independently extracted by two researchers and cross-checked after extraction to ensure accuracy. A HAMA score ≥ 7 was defined as the presence of anxiety symptoms, and a HAMD-17 score ≥ 8 was defined as the presence of depressive symptoms based on previously reported clinical thresholds [24].

Based on these predefined cutoff values, participants were classified into four groups: normal, anxiety, depression, and anxiety–depression symptom co-occurrence. The normal group was defined as participants without anxiety or depressive symptoms, corresponding to HAMA < 7 and HAMD-17 < 8. The anxiety group included participants with anxiety symptoms only (HAMA ≥ 7 and HAMD-17 < 8). The depression group included participants with depressive symptoms only (HAMA < 7 and HAMD-17 ≥ 8). The anxiety–depression symptom co-occurrence group included participants meeting criteria for both anxiety and depressive symptoms (HAMA ≥ 7 and HAMD-17 ≥ 8).

### 2.4. Statistical Analysis

Statistical analyses were performed using R version 4.3.2. Continuous variables are presented as mean ± standard deviation (SD) and compared using analysis of variance (ANOVA) or the Kruskal–Wallis test, as appropriate. Categorical variables are expressed as n (%) and compared using the chi-square test or Fisher’s exact test when appropriate. Multivariable linear regression analyses were conducted to assess the associations between VRF burden (defined as the number of VRFs) and HAMA and HAMD-17 scores, with separate models fitted for each outcome. Both models were adjusted for age, sex, marital status, BMI, and years of education. Regression coefficients (B) and 95% confidence intervals (CIs) were reported. Potential nonlinearity in the associations of VRF burden with HAMA and HAMD-17 scores was assessed by adding a quadratic term for VRF burden to the corresponding multivariable linear regression models.

For logistic regression analyses, anxiety–depression symptom co-occurrence was coded as 1 and the normal, anxiety, and depression groups as 0. Univariable logistic regression analyses were first performed to examine factors associated with anxiety–depression symptom co-occurrence. A primary multivariable logistic regression model was then constructed to examine the association between VRF burden and symptom co-occurrence, adjusting for sex, age, marital status, BMI, and years of education. A multinomial logistic regression was additionally performed using the four symptom groups as the outcome, with the normal group as the reference category, adjusting for the same covariates. In a separate multivariable model, all eight prespecified VRFs were entered simultaneously, with adjustment for the same covariates, to examine the associations of individual VRFs with symptom co-occurrence. VRF burden and its component risk factors were not included simultaneously in the same model.

Sensitivity analyses were conducted using a risk-factor-only score excluding CHD, CI, and TIA. This model was adjusted for sex, age, marital status, BMI, and years of education, with CHD, CI, and TIA additionally entered as separate variables in a further analysis. Odds ratios (ORs) and 95% CIs were reported for all logistic regression models. Regression analyses and statistical tests were performed primarily using the stats and nnet packages, with car and ResourceSelection used for model diagnostics. Graphical outputs were generated using ggplot2 and ggrepel. All tests were two-tailed, and *p* < 0.05 was considered statistically significant.

## 3. Results

### 3.1. Demographic and Clinical Characteristics of the Population

A total of 381 participants were included in the study, comprising 81 individuals in the normal group, 83 in the anxiety group, 61 in the depression group, and 156 in the anxiety–depression symptom co-occurrence group. Comparisons of demographic and clinical characteristics among the four groups are presented in Table 1.

Significant between-group differences were observed in sex distribution (*p* < 0.001), with the normal and depression groups predominantly male, whereas females were more frequently represented in the anxiety group. Smoking status also differed significantly across groups (*p* = 0.001), with the highest prevalence of current smoking observed in the depression group and the lowest in the anxiety group. No significant differences were found in age, marital status, BMI, or years of education (all *p* > 0.05). Regarding lifestyle factors, the prevalence of PI did not differ significantly among groups (*p* = 0.108), although it was highest in the co-occurrence group. Among VRFs, significant group differences were identified for diabetes mellitus (*p* < 0.001), hyperlipidemia (*p* = 0.043), and CI (*p* = 0.008). Participants in the co-occurrence group exhibited the highest prevalence of hypertension and hyperlipidemia, whereas diabetes was most common in the anxiety group. The prevalence of hypertension, CHD, and TIA did not differ significantly among groups (all *p* ≥ 0.05). VRF burden differed significantly among groups (*p* < 0.001), with the lowest values observed in the normal group and the highest value in the symptom co-occurrence group.

### 3.2. Neuropsychological Characteristics and Sex Distribution Across Groups

HAMA and HAMD-17 scores differed significantly among the four groups (both *p* < 0.001; Supplementary Table S1). The symptom co-occurrence group had the highest HAMA scores, followed by the anxiety group, whereas lower scores were observed in the normal and depression groups. HAMD-17 scores were highest in the depression and symptom co-occurrence group, with lower scores in the normal and anxiety groups.

Sex distribution also differed significantly across groups (*p* < 0.001; Table 1). As shown in Supplementary Figure S1, the distribution of emotional symptom groups varied by sex. Among males, the symptom co-occurrence group accounted for the largest proportion (33.9%), followed by the normal (27.3%), depression (22.9%), and anxiety (15.9%) groups. Among females, the symptom co-occurrence group was also the most common (53.7%), followed by the anxiety (32.4%), normal (10.3%), and depression (3.7%) groups. Overall, symptom co-occurrence represented the largest proportion in both sexes, while anxiety accounted for a greater proportion among females and depression for a greater proportion among males.

### 3.3. Associations Between VRF Burden and Emotional Symptoms

**Figure 1:**
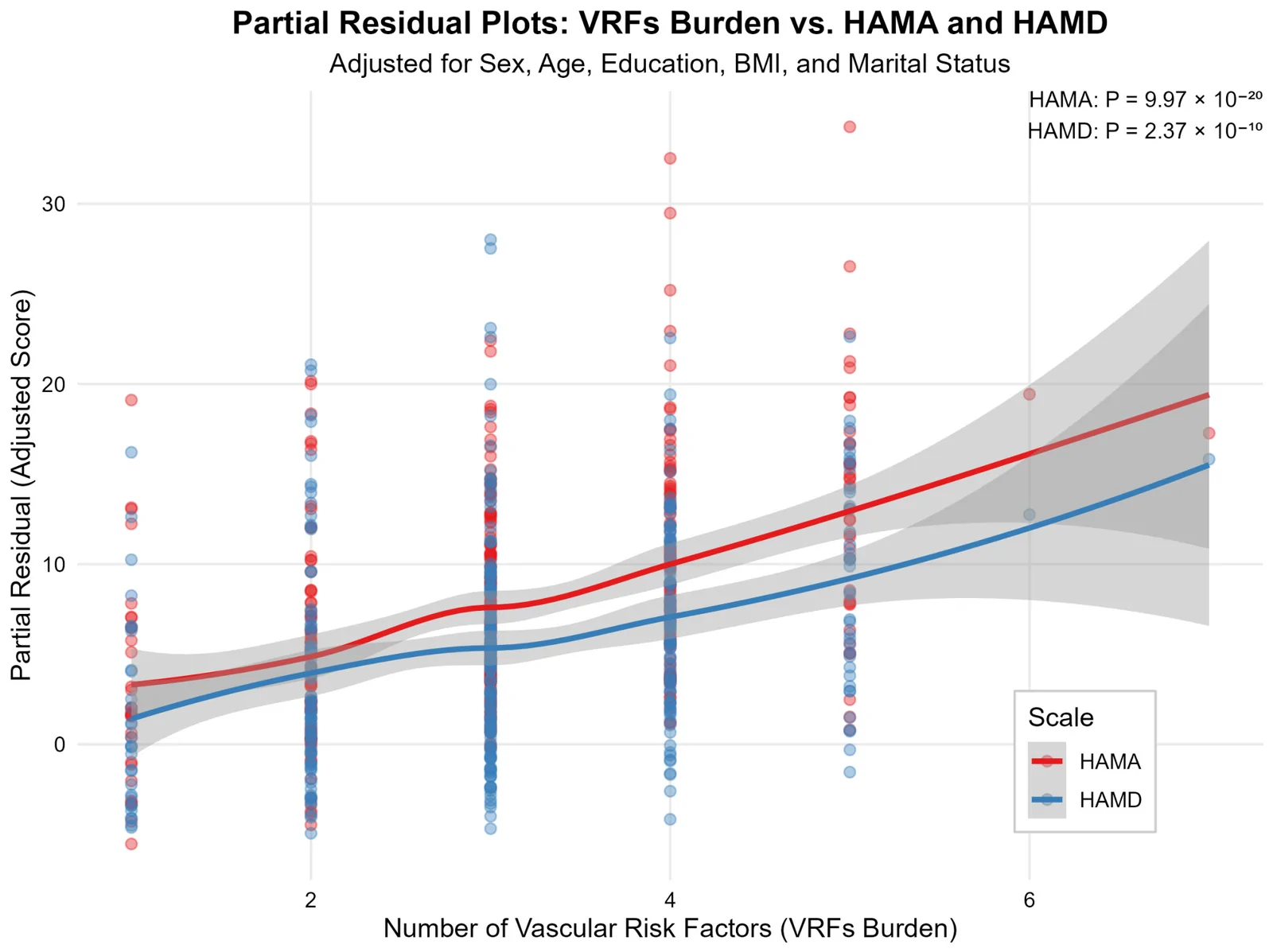
Trends in HAMA and HAMD-17 scores across different VRF burden levels. HAMA and HAMD-17 scores are shown across increasing levels of VRF burden. HAMA, Hamilton Anxiety Rating Scale; HAMD-17, 17-item Hamilton Depression Rating Scale; VRF burden, vascular risk factor burden.

As illustrated in Figure 1, greater VRF burden was associated with higher adjusted HAMA and HAMD-17 scores. In multivariable linear regression analyses adjusting for age, sex, years of education, BMI and marital status, VRF burden was positively associated with both HAMA and HAMD-17 scores (HAMA: B = 2.549, 95% CI: 2.028–3.070, *p* < 0.001; HAMD-17: B = 1.802, 95% CI: 1.258–2.346, *p* < 0.001). Female sex was independently linked to higher HAMA scores (B = 6.815, 95% CI: 5.596–8.035, *p* < 0.001) but not to HAMD-17 scores (B = 0.985, 95% CI: −0.289 to 2.258, *p* = 0.129). Age was not significantly associated with either outcome (HAMA: *p* = 0.169; HAMD-17: *p* = 0.163). Years of education were negatively associated with HAMD-17 scores (B = −0.222, 95% CI: −0.422 to −0.021, *p* = 0.030), whereas no such relationship was observed for HAMA scores (*p* = 0.085). BMI was negatively associated with HAMA scores (B = −0.212, 95% CI: −0.383 to −0.041, *p* = 0.015), but not with HAMD-17 scores (*p* = 0.130). Marital status was not significantly associated with either outcome (both *p* > 0.05). Additional analyses incorporating a quadratic term for VRF burden showed no evidence of significant nonlinearity for either HAMA (*p* for quadratic term = 0.392) or HAMD-17 (*p* for quadratic term = 0.847) (Supplementary Table S2).

Model diagnostics showed departures from residual normality for both HAMA and HAMD-17 models (both Shapiro–Wilk *p* < 0.001). Heteroscedasticity was detected for the HAMA model (Breusch–Pagan *p* = 0.003), but not for the HAMD-17 model (*p* = 0.494). Sensitivity analyses using HC3 robust standard errors yielded similar results, with VRF burden remaining significantly associated with both HAMA and HAMD-17 scores (Supplementary Table S3).

### 3.4. Factors Associated with Anxiety–Depression Symptom Co-Occurrence

**Figure 2:**
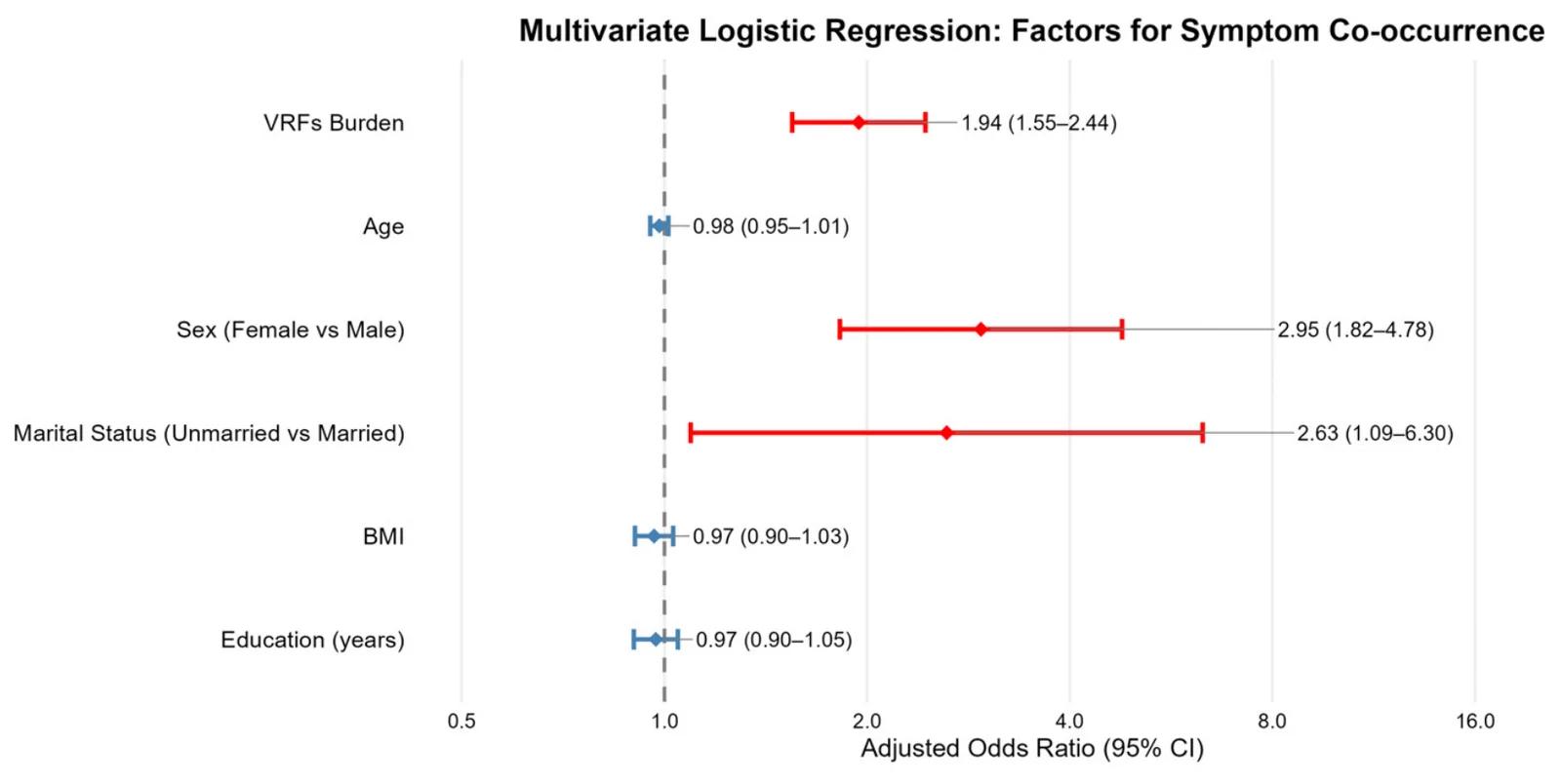
Multivariable logistic regression of VRF burden and anxiety–depression symptom co-occurrence. Forest plot showing the association between VRF burden and anxiety–depression symptom co-occurrence after adjustment for sex, age, marital status, BMI, and years of education. Odds ratios (ORs) and 95% confidence intervals (CIs) are shown. Greater VRF burden was associated with higher odds of anxiety–depression symptom co-occurrence. BMI, body mass index; VRF burden, vascular risk factor burden.

Univariable logistic regression analyses identified several factors associated with anxiety–depression symptom co-occurrence (Supplementary Figure S2). Higher VRF burden was associated with greater odds of symptom co-occurrence (OR = 1.701, 95% CI: 1.385–2.089, *p* < 0.001). Among individual factors, female sex (OR = 2.262, 95% CI: 1.473–3.471, *p* < 0.001), marital status (OR = 2.379, 95% CI: 1.082–5.232, *p* = 0.031), hypertension (OR = 1.662, 95% CI: 1.004–2.753, *p* = 0.048), diabetes (OR = 1.958, 95% CI: 1.291–2.968, *p* = 0.002), were significantly associated with symptom co-occurrence. No significant associations were observed for age, BMI, years of education, smoking status (current smoking), PI, hyperlipidemia, CHD, TIA, or CI (all *p* > 0.05). In the primary multivariable logistic regression model, greater VRF burden remained significantly associated with higher odds of anxiety–depression symptom co-occurrence after adjustment for sex, age, marital status, BMI, and years of education (adjusted OR = 1.944, 95% CI: 1.548–2.441, *p* < 0.001) (Figure 2).

To further examine the four symptom groups separately, multinomial logistic regression was performed using the normal group as the reference category. After adjustment for age, sex, marital status, BMI, and years of education, higher VRF burden was associated with greater odds of anxiety-only (OR = 2.855, 95% CI: 1.963–4.153, *p* < 0.001), depression-only (OR = 2.171, 95% CI: 1.501–3.141, *p* < 0.001), and anxiety–depression symptom co-occurrence (OR = 3.867, 95% CI: 2.712–5.516, *p* < 0.001) compared with the normal group (Supplementary Table S4).

**Figure 3:**
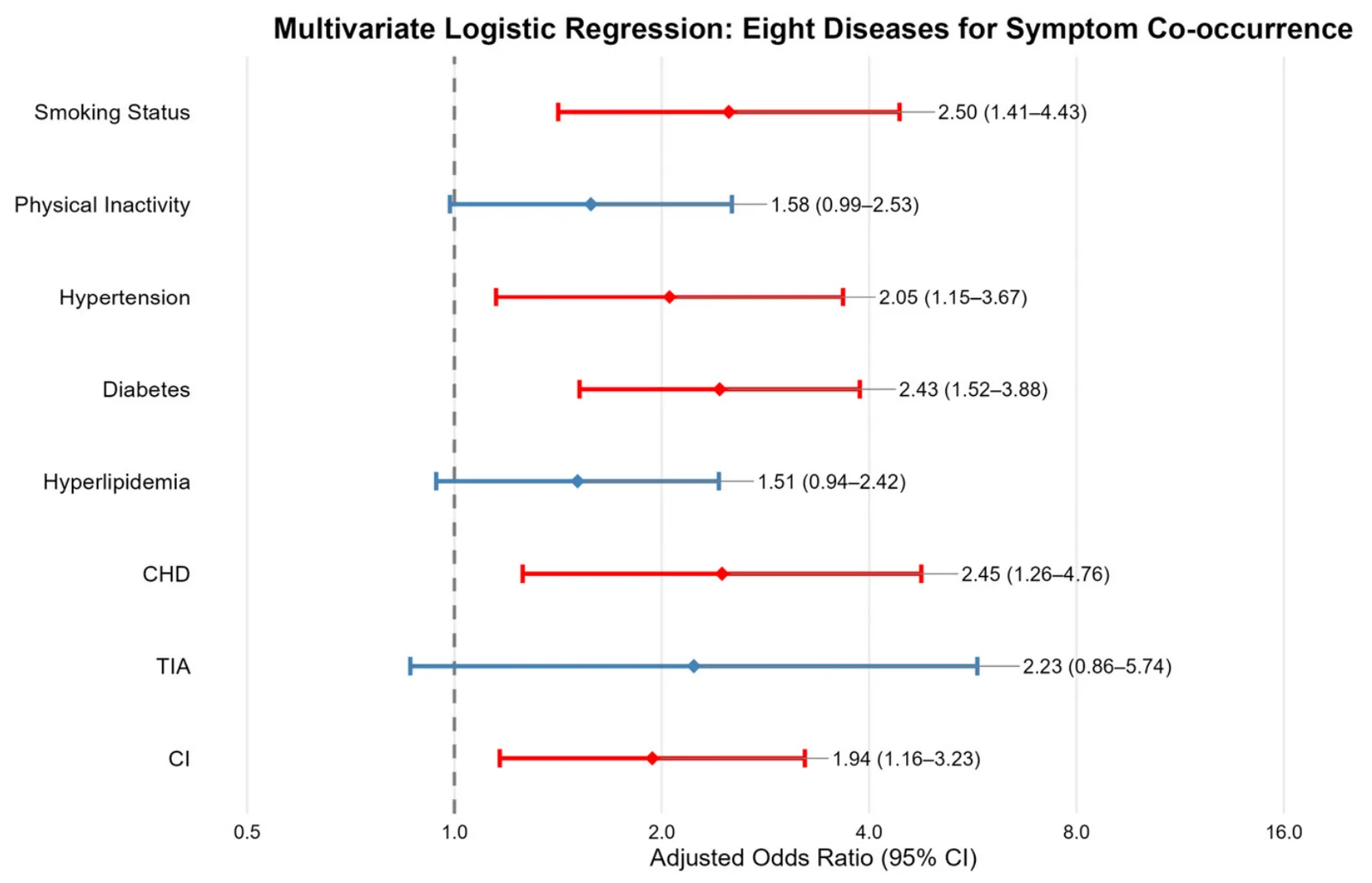
Adjusted associations of individual vascular risk factors with anxiety–depression symptom co-occurrence. Points indicate adjusted odds ratios (ORs), with horizontal lines representing 95% confidence intervals (CIs). All eight vascular risk factors were entered simultaneously into the multivariable logistic regression model, with adjustment for age, sex, marital status, BMI, and years of education. Participants without anxiety–depression symptom co-occurrence served as the reference group. BMI, body mass index; CHD, coronary heart disease. TIA, transient ischemic attack; CI, cerebral infarction.

In a separate multivariable logistic regression model (Supplementary Table S5 and Figure 3), all eight prespecified VRFs were entered simultaneously, with adjustment for age, sex, marital status, BMI, and years of education. Higher odds of anxiety–depression symptom co-occurrence were observed for current smoking (OR = 2.503, 95% CI: 1.415–4.428, *p* = 0.002), hypertension (OR = 2.053, 95% CI: 1.149–3.666, *p* = 0.015), diabetes (OR = 2.427, 95% CI: 1.520–3.877, *p* < 0.001), CHD (OR = 2.446, 95% CI: 1.256–4.762, *p* = 0.009), and CI (OR = 1.938, 95% CI: 1.164–3.226, *p* = 0.011), whereas PI, hyperlipidemia, and TIA were not significantly associated with the outcome.

Sensitivity analyses showed similar associations. The risk-factor-only score was associated with anxiety–depression symptom co-occurrence after adjustment for age, sex, marital status, BMI, and education (adjusted OR = 1.777, 95% CI: 1.401–2.255, *p* < 0.001), and after further adjustment for CHD, CI, and TIA (adjusted OR = 1.895, 95% CI: 1.478–2.430, *p* < 0.001; Supplementary Table S6).

## 4. Discussion

This study investigated the associations of VRF burden with anxiety symptoms, depressive symptoms, and anxiety–depression symptom co-occurrence among middle-aged and older adults with VRFs. Three main findings emerged. First, anxiety–depression symptom co-occurrence was the most common emotional symptom pattern in this clinical sample. Second, greater VRF burden was associated with more severe anxiety and depressive symptoms and with higher odds of anxiety–depression symptom co-occurrence after multivariable adjustment. Third, among individual VRFs, current smoking, hypertension, diabetes, CHD, and CI were associated with higher odds of symptom co-occurrence after adjustment. These findings suggest that cumulative VRF burden may be relevant to both emotional symptom severity and symptom co-occurrence.

Previous studies have mainly focused on individual VRFs and emotional symptoms, whereas the relationship between cumulative VRF burden and emotional health remains less clear. Anxiety and depressive symptoms are common in individuals with cardiovascular and metabolic conditions and are associated with poorer quality of life and adverse clinical outcomes [25,26,27]. Population-based HUNT data similarly showed frequent emotional symptoms among adults with cardiovascular disease and diabetes, with associations varying by sex and disease status [16]. Individual VRFs, including hypertension, diabetes, obesity, and smoking, have also been linked to greater anxiety and depressive symptom burden [5,28]. However, individual factors may not fully capture cumulative vascular risk. Assessing overall VRF burden may therefore provide a more comprehensive view of the relationship between vascular and emotional health.

In the present study, greater VRF burden was associated with more severe anxiety and depressive symptoms, consistent with emerging evidence on cumulative vascular risk. In the English Longitudinal Study of Aging, higher VRF burden was associated with higher levels of depressed mood in middle-aged and older adults [29], while a longitudinal UK Biobank study found a modest association with later depressive symptoms, without evidence of mediation by white matter microstructural changes [30]. These findings are broadly compatible with the vascular depression hypothesis, which proposes that cerebrovascular disease and vascular risk may contribute to depressive symptoms through disruption of neural circuits involved in mood regulation [31]. Longitudinal evidence further suggests that vascular burden and depressive symptoms may influence each other over time [32]. However, this framework was developed primarily for depression and does not fully explain anxiety or anxiety–depression symptom co-occurrence. The findings therefore extend previous work by suggesting that cumulative VRF burden may be relevant to a broader spectrum of emotional symptoms. Potential pathways may include endothelial dysfunction, altered cerebral perfusion, inflammation, oxidative stress, autonomic dysregulation, and other neurobiological or behavioral processes [8,9,10,33,34], although these mechanisms were not directly examined in the present study.

Anxiety–depression symptom co-occurrence was particularly prominent in this sample. Compared with isolated anxiety or depressive symptoms, comorbidity is generally associated with greater symptom burden and functional impairment [19,35]. In this study, the symptom co-occurrence group had relatively high VRF burden and emotional symptom severity, and greater VRF burden remained associated with higher odds of symptom co-occurrence after multivariable adjustment. These findings suggest that cumulative vascular burden may be relevant not only to the severity of individual emotional symptoms but also to their co-occurrence. This may be particularly relevant in general medical settings, where emotional, cognitive, sleep, and vascular problems often coexist rather than presenting as a single clearly defined psychiatric syndrome.

Several individual factors were also associated with anxiety–depression symptom co-occurrence. Female sex was associated with higher odds of symptom co-occurrence, consistent with previous studies showing a greater burden of anxiety symptoms among women [16,36,37]. In the model including all eight VRFs, current smoking, hypertension, diabetes, CHD, and CI were associated with higher odds of symptom co-occurrence after multivariable adjustment. These associations may reflect overlapping demographic, vascular, metabolic, and lifestyle-related influences on emotional health [38,39,40,41,42]. The association with current smoking should be interpreted cautiously, as it emerged only after multivariable adjustment.

These findings may have clinical relevance for general medical and neuropsychological settings, where emotional symptoms can remain underrecognized when attention is focused primarily on cardiovascular, metabolic, neurological, or cognitive concerns. Assessment of cumulative VRF burden may provide useful clinical context for recognizing patients who warrant further evaluation of anxiety and depressive symptoms. This is consistent with recent multicenter evidence in ischemic stroke showing that vascular and neurological factors are important predictors of 3-month functional outcome in middle-aged and older patients with cerebrovascular disease [43]. VRF burden should not be considered a psychiatric screening tool, but it may support more integrated consideration of vascular and emotional health.

This study has several limitations. First, this single-center cross-sectional study may have limited generalizability and cannot establish temporal or causal relationships. Reverse causality and bidirectional associations are possible: emotional symptoms may influence smoking or physical inactivity, whereas established vascular conditions such as CHD or cerebral infarction may contribute to anxiety or depressive symptoms. Because the VRF burden score included both conventional risk factors/lifestyle exposures and established vascular conditions, the direction of these associations cannot be determined. Referral and selection patterns may also have influenced the observed distributions. Second, anxiety and depressive symptoms were defined using HAMA and HAMD-17 thresholds rather than structured psychiatric interviews and therefore represent symptom-based rather than diagnostic categories. The binary logistic regression also combined the normal, anxiety-only, and depression-only groups into a heterogeneous reference category, which may limit interpretation. Previous or current anxiety or depressive disorders and psychotropic medication use were not systematically recorded and could not be adjusted for. Third, residual confounding from medication use, socioeconomic status, sleep disturbance, disease control, and other psychosocial factors cannot be excluded. Fourth, VRF burden was calculated as an unweighted count and did not account for differences in severity, duration, treatment status, or relative clinical impact. Sensitivity analyses using a risk-factor-only score showed similar associations, although the composite nature of the score should still be considered when interpreting the findings. Finally, no neuroimaging or biological markers were assessed, so potential mechanisms remain speculative.

Overall, cumulative VRF burden may be relevant to both emotional symptom severity and anxiety–depression symptom co-occurrence. Longitudinal studies are needed to clarify the temporal relationships underlying these associations.

## 5. Conclusions

Anxiety–depression symptom co-occurrence was common among individuals with VRFs. Greater VRF burden was associated with more severe anxiety and depressive symptoms and with higher odds of symptom co-occurrence after multivariable adjustment. Current smoking, hypertension, diabetes, CHD, and CI were also associated with symptom co-occurrence in the individual-VRF model. These findings should be interpreted in the context of a selected single-center clinical sample and the cross-sectional design, which precludes causal or temporal inference. Prospective longitudinal studies are needed to clarify the temporal and potentially bidirectional relationships between vascular burden and emotional symptoms.

## Figures and Tables

**Table 1:** Demographic characteristics and group comparisons.

Variable	Normal N = 81	Anxiety N = 83	Depression N = 61	Co-Occurrence N = 156	*p* Value
Sex					<0.001
Male	67 (82.7%)	39 (47.0%)	56 (91.8%)	83 (53.2%)	
Female	14 (17.3%)	44 (53.0%)	5 (8.2%)	73 (46.8%)	
Age, years	63.35 ± 7.64	64.60 ± 6.95	62.03 ± 7.14	63.29 ± 7.13	0.157
Marital Status					0.178
Married	77 (95.1%)	78 (94.0%)	59 (96.7%)	139 (89.1%)	
Unmarried	4 (4.9%)	5 (6.0%)	2 (3.3%)	17 (10.9%)	
BMI	25.27 ± 3.61	23.92 ± 2.79	24.61 ± 2.87	24.29 ± 3.52	0.085
Education (years)	11.23 ± 2.93	11.90 ± 2.98	11.89 ± 2.98	11.24 ± 2.96	0.309
Smoking Status					0.001
No	64 (79.0%)	71 (85.5%)	35 (57.4%)	113 (72.4%)	
Yes	17 (21.0%)	12 (14.5%)	26 (42.6%)	43 (27.6%)	
PI					0.108
No	55 (67.9%)	45 (54.2%)	38 (62.3%)	82 (52.6%)	
Yes	26 (32.1%)	38 (45.8%)	23 (37.7%)	74 (47.4%)	
Hypertension					0.050
No	22 (27.2%)	17 (20.5%)	21 (34.4%)	28 (17.9%)	
Yes	59 (72.8%)	66 (79.5%)	40 (65.6%)	128 (82.1%)	
Diabetes					<0.001
No	69 (85.2%)	38 (45.8%)	38 (62.3%)	75 (48.1%)	
Yes	12 (14.8%)	45 (54.2%)	23 (37.7%)	81 (51.9%)	
Hyperlipidemia					0.043
No	48 (59.3%)	35 (42.2%)	32 (52.5%)	65 (41.7%)	
Yes	33 (40.7%)	48 (57.8%)	29 (47.5%)	91 (58.3%)	
CHD					0.162
No	74 (91.4%)	71 (85.5%)	56 (91.8%)	129 (82.7%)	
Yes	7 (8.6%)	12 (14.5%)	5 (8.2%)	27 (17.3%)	
TIA					0.394
No	75 (92.6%)	81 (97.6%)	56 (91.8%)	145 (92.9%)	
Yes	6 (7.4%)	2 (2.4%)	5 (8.2%)	11 (7.1%)	
CI					0.008
No	50 (61.7%)	49 (59.0%)	22 (36.1%)	75 (48.1%)	
Yes	31 (38.3%)	34 (41.0%)	39 (63.9%)	81 (51.9%)	
VRF burden	2.36 ± 0.97	3.10 ± 1.03	3.11 ± 1.03	3.44 ± 1.05	<0.001

Note: BMI, body mass index; PI, physical inactivity; CHD, coronary heart disease; TIA, transient ischemic attack; CI, cerebral infarction; VRF burden, vascular risk factor burden. VRF burden was calculated as the number of present VRFs (current smoking, PI, hypertension, diabetes, hyperlipidemia, CHD, TIA, and CI), with higher scores indicating greater VRF burden.

## Data Availability

The datasets presented in this article are not readily available because this data belongs to the Zhongnan Hospital of Wuhan University system, and the authors don’t have the right to share it.

## Supplementary Materials

The following supporting information can be downloaded at: https://www.mdpi.com/article/10.3390/brainsci16101058/s1, Figure S1: Distribution of four emotional symptom groups by sex; Figure S2: Forest plot of univariable logistic regression for anxiety—depression symptom co-occurrence; Table S1: HAMA and HAMD-17 Scores Across Groups; Table S2: Assessment of potential nonlinear associations between vascular risk factor burden and HAMA/HAMD-17 scores; Table S3: Model diagnostics and robust sensitivity analyses for HAMA and HAMD-17; Table S4: Multinomial logistic regression of symptom groups with the normal group as the reference; Table S5: Multivariable logistic regression for anxiety—depression comorbidity; Table S6: Sensitivity analysis of the association between vascular risk factor burden and anxiety—depression symptom co-occurrence; Table S7: Categorical analysis of the association between vascular risk factor burden and anxiety—depression symptom co-occurrence (reply the reviewer).
