# Supplementary material for: Cumulative Vascular Risk Factor Burden and Anxiety–Depression Symptoms Co-Occurrence: A Cross-Sectional Study
Source: Brain Sci. 2026 Sep 30;16(10):1058. doi: 10.3390/brainsci16101058 (PMC13702224; doi:10.3390/brainsci16101058)
Supplement: Supplementary file 1 [file brainsci-16-01058-s001.zip › brainsci-4545038-supplementary.pdf]

Supplementary Materials

Supplementary Table S1. HAMA and HAMD-17 Scores Across Groups

Variable	Normal<br>N = 81	Anxiety<br>N = 83	Depression<br>N = 61	Co-Occurrence<br>N = 156	*P value*
HAMA	3.25 ± 2.00	12.39 ± 6.03	4.18 ± 1.73	14.44 ± 5.94	< 0.001
HAMD-17	3.06 ± 2.37	4.06 ± 2.23	13.20 ± 5.47	13.22 ± 4.62	< 0.001

Note: Data are presented as mean ± standard deviation (SD). Group differences were assessed using the Kruskal–Wallis test. HAMA, Hamilton Anxiety Rating Scale; HAMD-17, 17-item Hamilton Depression Rating Scale.

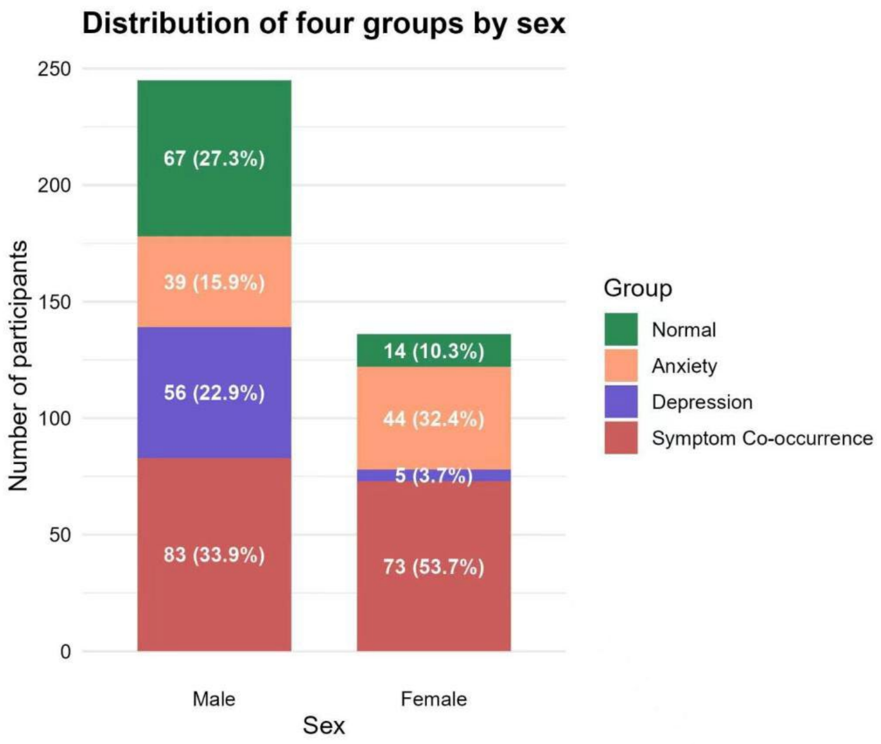

Supplementary Figure S1. Distribution of four emotional symptom groups by sex. Bars represent the proportions of participants classified as normal, anxiety, depression, or anxiety–depression symptom co-occurrence within each sex. Percentages were calculated separately for males and females.

Supplementary Table S2. Assessment of potential nonlinear associations between vascular risk factor burden and HAMA/HAMD-17 scores

Outcome	Linear term, β	SE	95% CI	*p* value	*p* for quadratic term
HAMA	2.549	0.265	2.028–3.070	< 0.001	0.392
HAMD-17	1.802	0.277	1.258–2.346	< 0.001	0.847

Note: Potential nonlinearity was assessed by adding a quadratic term for VRF burden (VRF burden²) to the multivariable linear regression models adjusted for age, sex, marital status, BMI, and education. No significant quadratic association was observed for either outcome.

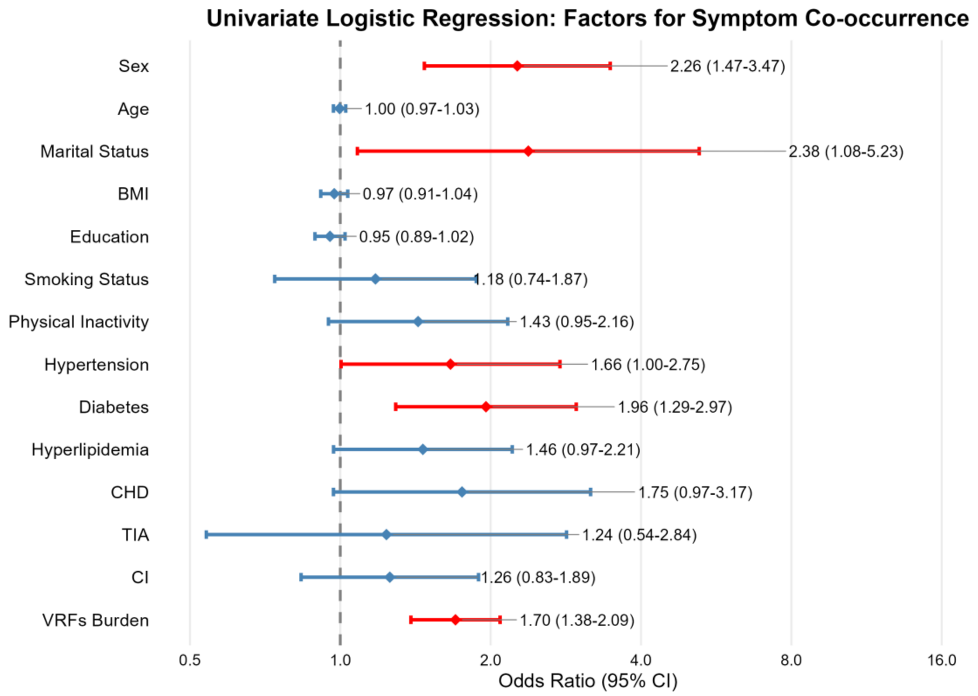

Supplementary Figure S2. Forest plot of univariable logistic regression for anxiety–depression symptom co-occurrence. Points indicate odds ratios (ORs), with horizontal lines representing 95% confidence intervals (CIs). Participants without anxiety–depression symptom co-occurrence served as the reference group. BMI, body mass index; CHD, coronary heart disease; TIA, transient ischemic attack; CI, cerebral infarction; VRF burden, vascular risk factor burden.

Supplementary Table S3. Model diagnostics and robust sensitivity analyses for HAMA and HAMD-17

Outcome	Breusch–Pagan *p*	Robust B (95% CI)	Robust *p*
HAMA	0.003	2.549 (1.966–3.132)	< 0.001
HAMD-17	0.494	1.802 (1.268–2.335)	< 0.001

Note: Robust estimates were obtained using HC3 heteroscedasticity-consistent standard errors. Models were adjusted for age, sex, marital status, BMI, and years of education.

Supplementary Table S4. Multinomial logistic regression of symptom groups with the normal group as the reference

Variable	Anxiety vs Normal, OR (95% CI)	*p*_value	Depression vs Normal, OR (95% CI)	*p*_value	Co-occurrence vs Normal, OR (95% CI)	*p*_value
VRF burden	2.855 (1.963–4.153)	< 0.001	2.171 (1.501–3.141)	< 0.001	3.867 (2.712–5.516)	< 0.001
Female (vs. male)	11.047 (4.812–25.361)	< 0.001	0.703 (0.228–2.168)	0.539	9.248 (4.223–20.251)	< 0.001
Age (per year)	1.011 (0.963–1.061)	0.652	0.973 (0.927–1.021)	0.260	0.980 (0.938–1.024)	0.364
Unmarried status (vs. married)	1.171 (0.243–5.640)	0.844	1.183 (0.192–7.267)	0.856	3.195 (0.816–12.510)	0.095
BMI (per kg/m²)	0.840 (0.753–0.936)	0.002	0.926 (0.827–1.038)	0.187	0.872 (0.791–0.961)	0.006
Education (per year)	1.124 (0.999–1.265)	0.052	1.082 (0.960–1.219)	0.196	1.045 (0.938–1.164)	0.425

Note: Multinomial logistic regression was performed using the four symptom groups as the outcome, with the normal group as the reference

category. The model included VRF burden and was adjusted for age, sex, marital status, BMI, and years of education. OR, odds ratio; CI, confidence interval; BMI, body mass index; VRF, vascular risk factor.

Supplementary Table S5. Multivariable logistic regression for anxiety–depression comorbidity.

Variable	OR (95% CI)	*p*_value
Female (vs. male)	3.48 (2.00–6.06)	< 0.001
Age (per year)	0.98 (0.95–1.01)	0.231
Unmarried status (vs. married)	2.68 (1.10–6.52)	0.030
BMI (per kg/m²)	0.96 (0.90–1.03)	0.290
Education (per year)	0.97 (0.90–1.05)	0.453
Current smoking (vs. no)	2.50 (1.42–4.43)	0.002
Physical inactivity (vs. no)	1.58 (0.99–2.53)	0.058
Hypertension (vs. no)	2.05 (1.15–3.67)	0.015
Hyperlipidemia (vs. no)	1.51 (0.94–2.42)	0.088
Diabetes (vs. no)	2.43 (1.52–3.88)	< 0.001
CHD (vs. no)	2.45 (1.26–4.76)	0.009
TIA (vs. no)	2.23 (0.86–5.74)	0.098
CI (vs. no)	1.94 (1.16–3.23)	0.011

Note: All eight prespecified vascular risk factors were entered simultaneously into the multivariable logistic regression model, with adjustment for age, sex, marital status, BMI, and years of education. OR, odds ratio; CI, confidence interval; BMI, body mass index; CHD, coronary heart disease; TIA, transient ischemic attack; CI, cerebral infarction.

Supplementary Table S6. Sensitivity analysis of the association between vascular risk factor burden and anxiety − depression symptom co-occurrence

Ananlysis	Variable	Adjusted OR (95% CI)	*p* value
Primary model	8-factors VRF burden	1.94 (1.55–2.44)	< 0.001
Sensitivity analysis 1	Risk-factor-only score	1.78 (1.40–2.26)	< 0.001
Sensitivity analysis 2	Risk-factor-only score	1.90 (1.48–2.43)	< 0.001
	CHD	2.46 (1.27–4.77)	0.008
	CI	1.93 (1.18–3.18)	0.01
	TIA	2.17 (0.84–5.58)	0.108

Note: The 8-factor VRF burden score included current smoking, physical inactivity, hypertension, diabetes, hyperlipidemia, CHD, TIA, and CI. The risk-factor-only score excluded CHD, TIA, and CI. All models were adjusted for age, sex, marital status, BMI, and years of education; sensitivity analysis 2 additionally included CHD, TIA, and CI as separate covariates. OR, odds ratio; CI, confidence interval; VRF, vascular risk factor; BMI, body mass index; CHD, coronary heart disease; TIA, transient ischemic attack; CI, cerebral infarction.

Supplementary Table S7. Categorical analysis of the association between vascular risk factor

burden and anxiety–depression symptom co-occurrence

VRF burden category	Adjusted OR (95% CI)	*p* value
1	Reference	—
2	1.69 (0.54–5.30)	0.366
3	4.90 (1.67–14.33)	0.004
4	7.85 (2.57–24.02)	< 0.001
≥5	12.96 (3.77–44.51)	< 0.001

Note: VRF burden was categorized by the number of vascular risk factors present. Observed scores ranged from 1 to 7; because only one participant had a score of 6 and one had a score of 7, scores of 5–7 were combined as ≥5. Logistic regression was performed with anxiety–depression symptom co-occurrence as the outcome and VRF burden of 1 as the reference category, adjusting for age, sex, marital status, BMI, and years of education. Adjusted ORs and 95% CIs represent the odds of symptom co-occurrence relative to the reference group. The eight prespecified VRFs were current smoking, physical inactivity, hypertension, diabetes, hyperlipidemia, coronary heart disease, transient ischemic attack, and cerebral infarction. This categorical analysis was performed to assess the robustness of the primary model, which treated VRF burden as a continuous variable.
